# The significance of thymoquinone administration on liver toxicity of diazinon and cholinesterase activity; a recommendation for prophylaxis among individuals at risk

**DOI:** 10.1186/s12906-022-03806-8

**Published:** 2022-12-05

**Authors:** Gholam-Hassan Danaei, Arian Amali, Mohammad Karami, Mohammad-Bagher Khorrami, Bamdad Riahi-Zanjani, Mahmood Sadeghi

**Affiliations:** 1 Imam Reza Hospital, Mashhad University of Medical Sciences, Mashhad, Iran; 2 Student Research Committee, Paramedical Department, Islamic Azad University, Mashhad Branch, Mashhad, Iran; 3 Department of Pharmacology and Toxicology, School of Pharmacy, Mazandaran University of Medical Sciences, Sari, Iran; 4 Social Security Organization, 17Th Shahrivar Hospital, Mashhad, Iran; 5 Medical Toxicology Research Center (MTRC), Mashhad University of Medical Sciences, Mashhad, Iran; 6 Medical Toxicology and Drug Abuse Research Center (MTDRC), Birjand University of Medical Sciences, Birjand, Iran

**Keywords:** Thymoquinone, Diazinon, Supplement therapy, Antioxidant, Hepatotoxicity, Cholinesterase

## Abstract

**Background:**

Diazinon (DZN), a widely used chemical herbicide for controlling agricultural pests, is an important organophosphorus pesticide and an environmental pollutant which induces toxic effects on living organisms during long-term exposure. Thymoquinone (TQ) is a phytochemical bioactive compound with antioxidant and anti-inflammatory properties. We aimed to evaluate the protective effects of TQ against DZN-induced hepatotoxicity through alleviating oxidative stress and enhancing cholinesterase (ChE) enzyme activity.

**Methods:**

Rats were randomly divided into six groups (*n* = 8); a negative control group receiving corn oil; a group only receiving DZN (20 mg/kg/day); a group treated with TQ (10 mg/kg/day), and three treatment groups as TQ + DZN, receiving different doses of TQ (2.5, 5, and 10 mg/kg/day). All experimental animals were orally treated for 28 consecutive days. The levels of superoxide dismutase (SOD), glutathione (GSH), malondialdehyde (MDA), alanine transaminase (ALT), aspartate aminotransferase (AST), alkaline phosphatase (ALP), and lactic acid dehydrogenase (LDH) were determined. In addition, ChE activity and histopathological changes were evaluated.

**Results:**

The results showed that DZN decreased GSH level (*p* < 0.01) and SOD activity (*p* < 0.01) in parallel to an increase in MDA level (*p* < 0.01) and increased the activity of AST, ALT, ALP, and LDH (*p* < 0.01) in comparison to the negative control group. Our findings demonstrated that TQ administration could diminish hepatotoxicity and reduce oxidative damage in DZN-treated rats, which could be linked to its antioxidant and free radical scavenging properties. It was also observed that TQ 10 mg/kg remarkably increased the activity of acetylcholinesterase, butyrylcholinesterase, and SOD enzymes, elevated GSH, decreased MDA, and reduced pathological alternations of the liver induced by DZN.

**Conclusion:**

Thymoquinone 10 mg/kg increased the activity of plasma and blood cholinesterases and reduced DZN-induced alternations of the liver. Improvement of butyryl- and acetylcholinesterase activity suggests that maybe TQ supplement could be beneficial as pre-exposure prophylaxis among farm workers spraying pesticides.

## Background

Extensive quantities of organophosphate (OP) pesticides are used in many developing countries to maintain food supplies [1, 2]. The excessive use of OPs is a global issue that causes vast environmental pollution, which can affect humans and other species’ health [3]. There are various types of OPs with different brands in the market which are classified based on toxicity potential. Among the OPs, diazinon (DZN) is applied more than others due to its less toxic properties on mammals to control and protect agricultural and horticultural products from pests. The main environmental advantage of this compound could be photochemical inactivation by sunlight, which limits the chronicity of environmental damage and makes it a favored product for homeowners to be used for lawns, gardens, and interior spaces. The entrance of DZN into food chains and its accumulation in the body may cause adverse reactions such as heart, pancreas, kidney, brain, and liver dysfunctions [4, 5]. Health Canada's screening values has identified a drinking water screening limit of 0.015 mg/L (15 µg/L) for DZN. Besides, the maximum residue limits established for DZN in foods is 0.75 ppm [6].

Cholinesterase (ChE) is an important enzyme of the functioning of the nervous system which might be modulated by chemical compounds and pharmaceuticals [7]. The foremost mechanism of action of DZN (and its metabolites) is the inhibition of acetylcholinesterase (AChE), which causes an increase in acetylcholine neurotransmitter in synaptic space, over-stimulation of cholinergic receptors at the postsynaptic membrane, and consequent muscarinic and nicotinic complications [8, 9]. DZN-induced phosphorylation of the esteratic site of AChE produces irreversible inhibition of the enzyme unless a reactivator such as pralidoxime be used. However, the oxime antidote can only reactivate the enzyme, if given in time, before loss of an alkyl group from phosphorylated AChE could age the enzyme [10]. Previous studies have reported that the toxic effects of DZN and its potent metabolites are not limited to the hyperactivity of cholinergic receptors, but it also induces oxidative stress through generating reactive oxygen species (ROS) [11–13]. ROS are chemically electrophile, reactive molecules [14–16], and their increase can result in the disturbance of the balance between the production of free radicals and antioxidant defenses, which causes oxidative damage to cellular structures [17–20]. It has been revealed that bioactive molecules and natural antioxidants can effectively scavenge free radicals and diminish damages associated with oxidative stress. Currently, natural compounds have attracted many researchers to counteract certain types of toxins and pathogenic factors [21, 22]. *Nigella sativa*, also known as black seed or black cumin, is an annual flowering plant that belongs to the family Ranunculaceae. As a natural compound with remedial properties, it has been used for a variety of health conditions in many Middle Eastern and Asian countries for centuries. The seeds are traditionally used for the treatment of pulmonary, cardiovascular, and gastrointestinal diseases, as well as inflammation, diabetes, nervous disorders, rheumatism, and various cancers [23]. There are many organic, mineral, and vitamin compounds in *N. sativa* seeds, which each of these plant's constituents can exhibit multiple pharmacological or therapeutic effects.

Thymoquinone (TQ; 2-isopropyl-5-methylbenzo-1, 4-quinone) is the most bioactive component of the volatile oil of *N. sativa* seeds that has potent pharmacological properties including antioxidant, anti-inflammatory and immunomodulatory, antimicrobial, neuroprotective, nephroprotective as well as hepatoprotective effects along with other beneficial effects [24–27]. Administration of *N. sativa* oil can possess hepatoprotective activity against carbon tetrachloride (CCl_4_) induced hepatotoxicity in male Wistar rats which provides a rationale to the medicinal use of this herbal supplement [28]. TQ as the main constituent of *N. sativa* has the potential benefits in the prevention of the onset and progression of cisplatin-induced hepatotoxicity [29]. TQ as a relatively safe compound and the combination with Vit D has anti-fibrogenic properties and hepato-protective effects against previously established liver fibrosis [30]. TQ administration can shows lower the levels of oxidative stress and elevation of the total antioxidant capacity indices in diabetic rats [31]. Furthermore, TQ treatment may improve AChE activity in by chlorpyrifos toxicity and alleviate neuronal injuries and oxidative stress [32, 33]. In this context, earlier studies have demonstrated that presence of many quinones especially thymoquinone as natural antioxidants in *N. sativa* and its extract is responsible for the improvement of oxidative stress and the subsequent organ damages induced by some xenobiotic and chemicals. Recent researches on TQ in rats and mice have shown that this bioactive compound has cardioprotective, neuroprotective, and nephroprotective effects based on its anti-inflammatory properties [34–40]. Some animal studies have demonstrated that TQ has therapeutic benefits against brain injury, ovarian cyst formation, and tumor growth [34, 39, 41, 42]. The hepatoprotective effects of TQ have also been suggested in rat models so that TQ may inhibit oxidative damage and might be able to prevent liver lesions through prevention of free radicals damage, lipid peroxidation, and by improving antioxidant sources [39, 43, 44]. However, there is still a lacking of knowledge of this effect on liver and the activity of ChE enzymes. Therefore, in this investigation, the effectiveness of oral supplementation of TQ as a potent natural antioxidant against DZN-induced hepatotoxicity in rats was examined on liver, blood, and biochemical factors.

## Materials and methods

### Chemical

Diazinon (purity ≥ 96%) was purchased from Ariashimi Co., Iran. Thymoquinone (C_10_H_12_O_2_; CAS number 490–91-5), dithiobisnitrobenzoic acid (DTNB), and acetylthiocholine iodide (ATCI) were procured from Sigma-Aldrich, Germany. Corn oil was obtained from Zarrin, Iran. Morin Biochemical colorimetric kits and antioxidant kits were purchased from BioLabo and Diaclone, France.

### Animals and experimental design

All experiments were approved by the animal ethics committee of Mazandaran University of Medical Science, Sari, Iran (IR.MAZUMS.172319). Male Wistar rats weighing 150–200 g were obtained from the laboratory animal center of. The animals were acclimatized for 1 week prior to experimentation. The animals were maintained in propylene cages in an air-conditioned room (temperature 24 ± 3 °C; relative humidity 50 ± 10%; 12 h dark/light cycle), which had free access to standard diet pellets and drinking water ad-libitum.

The animals were randomly divided into six groups (8 rats in each group). Group 1 was considered to be the control group and received corn oil. Groups 2–6 received DZN (20 mg/kg/day), TQ (10 mg/kg/day), TQ (2.5 mg/kg/day) + DZN, TQ (5 mg/kg/day) + DZN, and TQ (10 mg/kg/day) + DZN, respectively. All animals were treated orally once a day for four weeks. Applied doses of DZN and TQ were selected for this experiment based on previous studies [9, 21]. Administration of TQ solution in corn oil was done by gavage. At the end of the treatment (28 days), the animals were sacrificed. Hence, an i.p. injection of the anesthetic cocktail including ketamine-xylazine-acepromazine (50–10-1.5 mg/kg, respectively) was used before scarifice. Then, blood samples were withdrawn through cardiac puncture for the examination of biochemical parameters and cholinesterase activity assay. Liver tissues were used to assess markers of oxidative stress and histopathological changes.

### Biochemical parameters measurement

Blood samples were collected into heparinized tubes and were centrifuged at 3000 rpm for 10 min at 4 °C, and plasma was separated to determine biochemical parameters, including alanine transaminase (ALT), aspartate aminotransferase (AST), alkaline phosphatase (ALP), and lactic acid dehydrogenase (LDH) using Biolabo kits according to the instructions of the manufacturer.

### Cholinesterase activity assay

The activity of acetylcholinesterase (AChE) in compact RBCs of treated rats was determined using the Ellman method [45]. Collected blood samples were centrifuged at 5000 rpm for 10 min at 4 °C. The compacted lower layer (RBCs) was isolated and washed thrice with cold normal saline. Then, the compact RBCs were suspended in distilled water and incubated with DTNB + ATCI containing guanidine sulfate as the plasma cholinesterase inhibitor at 37 °C for 10 min. The reaction was terminated by adding 0.002% hyamine (benzethonium chloride), and the absorbance was measured at 440 nm using a spectrophotometer (Shimadzu; Japan). The AChE activity of blood samples was calculated by multiplying the optical density (OD) of the samples by a converting factor of 17.68 U/ml/pack cell.

The serum cholinesterase activity was measured using a colorimetric commercial kit (Biorexfars; Iran).

### Assessment of oxidative stress markers

The biomarkers of oxidative stress were measured in liver tissue homogenate in all the experimental groups. The tissue samples were quickly removed and weighed. A part of the liver samples was minced into smaller pieces and homogenized gently for 2 min, and then the homogenates were centrifuged at 6000 rpm for 15 min at 4 °C. Levels of malondialdehyde (MDA), superoxide dismutase (SOD), and glutathione (GSH) in supernatant fractions were determined using commercial mouse kits (Zell Bio; Germany).

### Histopathological examination

For histopathological examination, a portion of the liver samples was immersed in 10% neutral buffered formalin. The tissues were routinely processed, and paraffin-embedded samples were cut into sections at 4 µm thickness and stained with hematoxylin–eosin (H&E). A semi-automatic microtome, RMT-SA3315 (Japan) was used to generate tissue slides.

### Statistical analysis

All values were expressed as Mean ± Standard Deviation for all groups. For comparison between groups, statistical analysis was performed using one-way ANOVA followed by the Tukey multiple comparison test. A *p*-value of < 0.05 was considered to be statistically significant.

## Results

### Biochemical parameters

As shown in Table 1, rats exposed to DZN had significantly increased levels of serum LDH (*p* < 0.01), AST (*p* < 0.001), ALT (*p* < 0.01), and ALP (*p* < 0.001) as compared to the control group. On the other hand, the DZN + 2.5 mg/kg TQ rat group recorded a similar increase in AST and ALP activity (*p* < 0.01 for both enzymes) in comparison with the control animals, whereas administration of TQ at the dose of 10 mg/kg significantly declined the activity of all four enzymes when compared to DZN-treated groups. Moreover, the rats receiving 5 mg/kg of TQ showed a significant decrease in ALP serum levels as compared to DZN-treated groups (*p* < 0.05).

**Table 1:** Effect of TQ on biochemical parameters of different groups of rats

Parameters	Control	DZN	TQ	TQ2.5 + DZN	TQ5 + DZN	TQ10 + DZN
ALT (U/L)	39.30 ± 5.70	59.70 ± 8.50^aa^	40.50 ± 7.20	54.70 ± 6.30	50 ± 3.90	42.20 ± 3.50^b^
AST (U/L)	92.20 ± 10.30	143.30 ± 16.70^aaa^	92 ± 10.20	129.80 ± 14.30	118.30 ± 19.60	102.80 ± 16.50^bb^
ALP (U/L)	145.0 ± 17.10	237.50 ± 37.40^aaa^	146.30 ± 16.20	204.50 ± 31	187.80 ± 24.70^b^	156.30 ± 15.60^bb^
LDH (U/L)	413.80 ± 51.40	626.30 ± 54.50^aa^	426.80 ± 108.60	592.60 ± 36.70	473.50 ± 74.30	422.5 ± 58.70^bb^

^a^*P* < 0.05

^aa^*P* < 0.01

^aaa^*P* < 0.001 indicate significant differences in comparison to control groups

^b^*P* < 0.05

^bb^*P* < 0.01 indicate significant differences in comparison to DZN-treated groups

### Cholinesterase activity

**Fig. 1:**
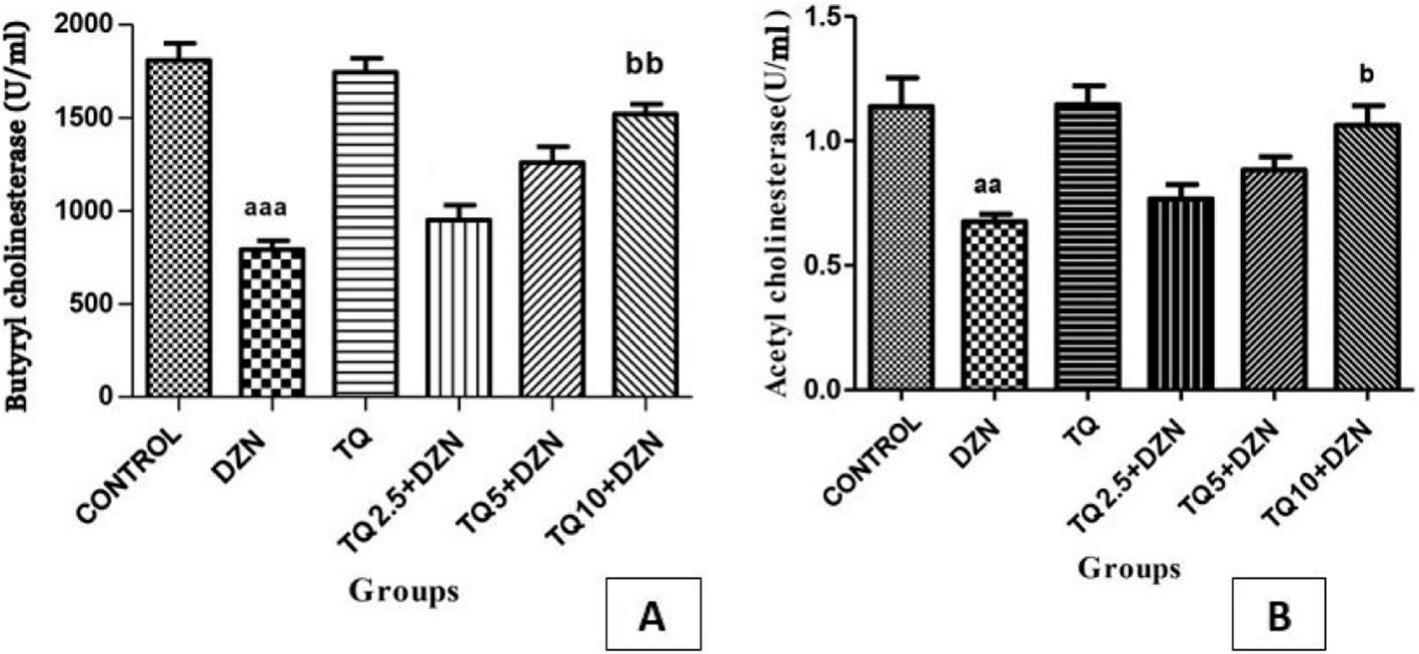
Effect of TQ on butyryl (1**A**) and acetyl (1**B**) cholinesterase activity of different groups of rats. **1A** ^aaa^*P* < 0.001 indicate significant differences in comparison to control groups. ^bb^*P* < 0.01 indicates significant differences in comparison to DZN-treated groups. **1B** ^aa^*P* < 0.01 indicate significant differences in comparison to control group. ^b^*P* < 0.05 indicates significant differences in comparison to DZN-treated groups

As demonstrated in Fig. 1, butyrylcholinesterase (plasma cholinesterase) and erythrocyte cholinesterase (RBC-cholinesterase) activity significantly decreased in the DZN-treated rats as compared to the corn oil control group (*p* < 0.001 and *p* < 0.01, respectively). Administration of TQ at the dose of 10 mg/kg remarkably increased the activity of the aforementioned enzymes as compared to DZN-treated rats, while groups that received TQ with lower doses (2.5 and 5 mg/kg) did not indicate any significant changes.

### Oxidant-antioxidant status

As shown in Table 2, oral administration of DZN caused a disturbance in the balance between the production of free radicals and the defense activity of antioxidants in rat liver tissue. DZN treatment resulted in a significant increase in MDA (*p* < 0.01) levels and a decrease in the level of GSH (*p* < 0.01) in comparison with the control group. There was a significant increase in the level of GSH (*p* < 0.01) and a decrease in the level of MDA (*p* < 0.05) in rats treated with DZN + TQ (10 mg/kg) as compared with the DZN–treated groups.

**Table 2:** Effect of TQ on redox status of tissue liver among different groups of rats

Parameters	Control	DZN	TQ	TQ2.5 + DZN	TQ5 + DZN	TQ10 + DZN
SOD (U/mL tissue)	27.20 ± 5.70	18.70 ± 6.20^aa^	31.5 ± 5.14	21.5 ± 4.90	23.30 ± 7.10	25.00 ± 6.20^b^
GSH (nmol/mg/p)	17.60 ± 1.08	10.90 ± 0.80^aa^	16.80 ± 1.2	12.20 ± 0.60	14.80 ± 0.80	15.90 ± 1.00 ^bb^
MDA (µmol/mL tissue)	3.75 ± 0.90	6.85 ± 10^aa^	3.86 ± 0.80	5.90 ± 0.59	5.12 ± 0.65	4.3 ± 0.3^b^

^aa^*P* < 0.01 indicates significant differences in comparison to control groups

^b^*P* < 0.05

^bb^*P* < 0.01 indicate significant differences in comparison to DZN-treated groups

### Histopathological examination

**Fig. 2:**
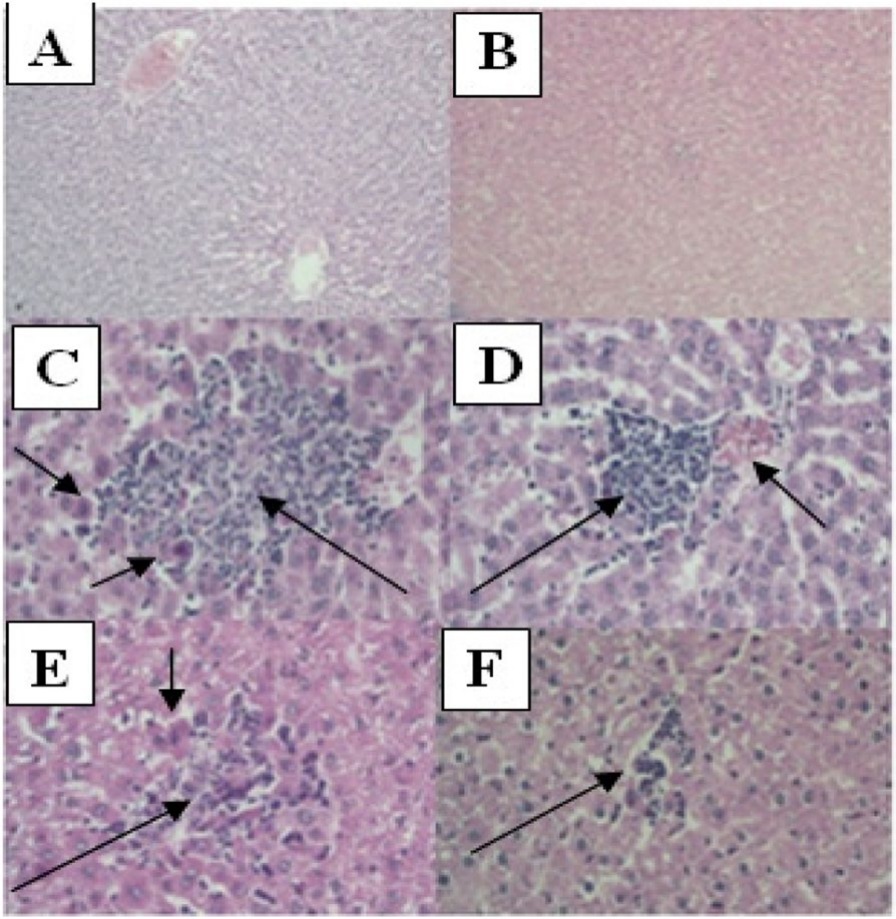
(**A** and **B**) Liver micrographs of control and TQ-treated groups (magnification ^*^100). (**C**) DZN treated group, (**D**, **E**, and **F**) DZN plus TQ at the doses of 2.5,5 and 10 mg/kg treated groups (magnification ^*^400). Necrosis showed by large arrow and apoptosis showed by small arrow

Histopathological findings related to the rats’ liver are portrayed in Table 3 and Fig. 2. DZN-treated groups showed various degrees of pathological lesions, including damage of liver structure, multifocal necrosis, apoptosis, hemorrhage, and edema when compared to the control group. Significant differences in histopathological characteristics were not observed in rats treated with the TQ dose of 2.5 and 5 mg/kg. In contrast, TQ at the dose of 10 mg/kg significantly reduced pathological alternations of the liver induced by DZN compared with the DZN group. Table 3 shows a dose–response gradient for the mitigating effect of TQ in the presence of the stressor DZN.

**Table 3:** Grading of the histopathological changes among different groups of rats

Histopathological findings	Control	DZN	TQ	TQ2.5 + DZN	TQ5 + DZN	TQ10 + DZN
Multifocal necrosis	0	+ + +	0	+ + +	+ +	+
Apoptotic body	0	+ +	0	+	+	0
Hemorrhage	0	+	0	0	0	0
Edema	0	+ +	0	+ +	+ +	0

## Discussion

The results of histopathological examinations, along with the activities of liver functional enzymes, indicated that subacute exposure to DZN could induce hepatotoxicity in rats. These alterations were accompanied by an increase in oxidative stress in liver tissue of the rats. Administration of TQ at the dose of 10 mg/kg remarkably ameliorated DZN-induced hepatotoxicity, reduced oxidative stress, and improved cholinesterase activity.

The signs and symptoms of acute OP poisoning have been well described, while the chronic effects of exposure to these compounds are not entirely clear. Many researchers postulate that the redox process in the organs of the body may be impaired due to OP toxic effects, thus leading to an increase in the level of lipid peroxidation [46–48]. As the enhanced generation of reactive oxygen species and induction of lipid peroxidation underlie many diseases, it is imperative to measure the effect of OP insecticides on the redox status of organs [48, 49], and it is equally vital to find protective mechanisms against pesticide-induced oxidative stress [48]. It is well understood that DZN increases the formation of ROS in tissues and erythrocytes, leading to oxidative stress [50, 51], which was confirmed in our study by the decrease in liver GSH level / SOD activity and an increase in MDA level. The present results support the hypothesis that oxidative stress and free radicals play an important role in DZN hepatotoxicity. One the other hand, TQ was found to be as potent antioxidant against DZN-induced hepatotoxicity.

Our findings showed that TQ and its metabolites, such as glutathione-dihydrothymoquinone and thymohydroquinone, as protective agents, were able to relieve oxidative stress induced by DZN due to having functional groups such as thiol (SH) and hydroxyl (OH) in their chemical structure [52]. The protective role of TQ can also be attributed to its potent phytochemical antioxidants that scavenge different types of oxygen radicals including superoxide anion, hydroxyl radical, and singlet molecular oxygen, and therefore, reduce ROS and oxidative stress levels [53]. Furthermore, as a quinone structure, TQ is able to cross the plasma membrane and thus reach the action-site in the intracellular organelles and scavenge free radicals and prevent them from causing oxidative damage to cellular components such as protein, DNA, and lipids [54]. Similar to our results, Maheswari et al., (2014) showed that while the level of ALT, AST and ALP increased by carbamazepine, administration of N-acetylcysteine, a known hepatoprotective drug, could decrease these parameters and increase the level of glutathione [55]. Similarly, N-acetyl-L-cysteine suppressed the increase in markers of liver functional state such as ALT, AST, and ALP and ameliorated GSH and SOD in liver damage induced by CCl_4_ [56].

OP toxicity is mainly attributed to the inhibition of acetylcholinesterase enzyme. Consistent with this fact, in our study, DZN-treated groups exhibited significant decreases in serum and RBC cholinesterase activity levels. On the other hand, TQ was found to have protective effects on both cholinesterases as our findings demonstrated an elevation in their activities when compared to DZN-treated groups. As an option, ROS and free radicals generated by DZN might disrupt the spatial conformation and integrity of cholinesterase, leading to lowered enzyme activity. Therefore, TQ as a potent antioxidant may scavenge and neutralize free radicals resulting in the improvement of enzyme inhibition. Apart from antioxidant capacity enhancement, TQ can reduce pro-inflammatory mediator TNF-α, revert the elevation of liver enzymes, and regulate pro and anti-apoptotic genes, and reduce the NF-kβ [57, 58]. To justify this phenomenon, we came across a study describing a reduction in cholinesterase activity of neuromuscular junction of the diaphragm among rats undergoing oxidative stress [59]. According to their findings, they considered that oxidative stress might decrease cholinesterase activity. In addition, TQ with an unknown mechanism might reactivate the inhibited enzymes. However, contrary to the present research, in a study performed by Hariri et al., TQ supplementation had no effects on cholinesterase activity [5].

Some hospitals reportedly provide complementary and alternative medicine therapy and there are also reports of individuals using traditional medicine service as well. Such services support traditional indigenous healers for management of some diseases [60, 61]. Since co-exposure to TQ leads to the higher activity of the cholinesterase enzyme of rats receiving DZN in comparison to the group with exposure to DZN alone (Fig. 1), and also considering that cholinesterase is a main target in the DZN toxicity, it is conceivable that TQ supplement can be taken as pre-exposure prophylaxis among farm workers spraying pesticides. Previous studies have also indicated the success of TQ supplementation against DZN toxicity in rats’ model of cardio- and hemato-toxicity [62, 63].

Of course, further studies are required to investigate the aforementioned implications and to determine the most appropriate dose and form of TQ usage.

## Conclusion

The findings of our study demonstrated that TQ and its metabolites were capable of countervailing DZN-induced hepatotoxicity in a dose-dependent manner and exerted the protective effects, probably through the deactivation of free radicals generated following exposure to DZN, to maintain the integrity of hepatocytes. TQ 10 mg/kg increased the activity of plasma and blood cholinesterases and reduced DZN-induced alternations of the liver. Improvement of butyryl- and acetylcholinesterase activity suggests that maybe TQ supplement could be beneficial as pre-exposure prophylaxis among farm workers spraying pesticides.

## Data Availability

The datasets used and analysed during the current study are available from the corresponding author on request.
